# Improved Prediction of Molecular Response to Pulling by Combining Force Tempering with Replica Exchange Methods

**DOI:** 10.1021/acs.jpcb.3c07081

**Published:** 2024-01-17

**Authors:** Yuvraj Singh, Glen M. Hocky

**Affiliations:** † Department of Chemistry, New York University, New York, New York 10003, United States; ‡ Simons Center for Computational Physical Chemistry, New York University, New York, New York 10003, United States

## Abstract

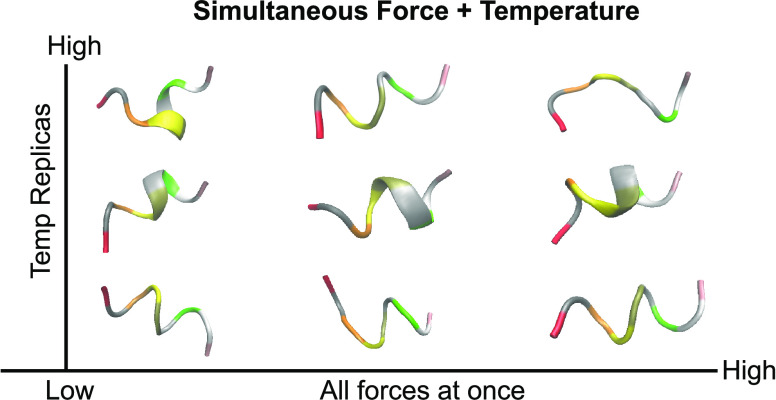

Small mechanical forces play important functional roles in many crucial cellular processes, including in the dynamic behavior of the cytoskeleton and in the regulation of osmotic pressure through membrane-bound proteins. Molecular simulations offer the promise of being able to design the behavior of proteins that sense and respond to these forces. However, it is difficult to predict and identify the effect of the relevant piconewton (pN) scale forces due to their small magnitude. Previously, we introduced the Infinite Switch Simulated Tempering in Force (FISST) method, which allows one to estimate the effect of a range of applied forces from a single molecular dynamics simulation, and also demonstrated that FISST additionally accelerates sampling of a molecule’s conformational landscape. For some problems, we find that this acceleration is not sufficient to capture all relevant conformational fluctuations, and hence, here we demonstrate that FISST can be combined with either temperature replica exchange or solute tempering approaches to produce a hybrid method that enables more robust prediction of the effect of small forces on molecular systems.

## 1 Introduction

Biological systems must have mechanisms for being able to sense and respond to mechanical forces from their environment and those that are generated internally through the action of molecular machines.^1−4^ Cells can employ proteins to sense and respond to these forces using a wide range of molecular mechanisms which we previously reviewed.^4^ Perhaps the simplest such mechanism is the use of a single disordered peptide domain at the locus of a mechanical process, whose change from a collapsed to an extended conformation with single piconewtons of force could be sufficient to change the behavior of a larger protein machine. This kind of behavior has been identified in polymerization factors called formins through a combination of in vitro and in vivo biochemistry with simple modeling,^5−9^ but a precise molecular mechanism for such behavior which explains the differences between homologous proteins in different species has not yet been shown.^8,9^

While these formin-disordered domains are very large and the effect of force on their activity is complex, the effect of a pulling force on simple peptides has been exploited for the development of molecular sensors termed tension sensor modules (TSMs).^10−12^ These TSMs consist of a short protein or peptide with donor and acceptor dye molecules that can undergo fluorescence resonance energy transfer (FRET) on the termini.^10,12^ Because FRET energy transfer is highly sensitive to distance, the FRET signal can be used to infer the distance between the ends of the molecule; this distance can be converted into a force through calibration experiments performed with molecular tweezers, if a specially selected molecule is chosen which does not exhibit hysteresis.^10^ Genetically encoded TSMs can then be used to measure the forces felt by certain proteins in living cells, such as those within focal adhesion complexes, which serve as the connection between the internal cytoskeleton and the exterior environment of a cell.^13,14^ These measurements were used to confirm the relevance of 1–20 pN forces in focal adhesion behavior.^15^ Through experimentation, different peptides or small proteins have been found that exhibit peak force sensitivity over different ranges.^10,11,16^ Our ultimate goal in this work is to advance molecular simulation approaches such that we can predict in silico the sensitivity of a disordered peptide sequence or small folded protein to pN scale forces.

Molecular dynamics simulations (MD) can reveal highly detailed molecular-level information about a wide range of biomolecular systems.^17,18^ To explore a biomolecule’s conformational landscape using a reasonable amount of computational expense, it is often necessary to employ enhanced sampling techniques that bias the system’s behavior in such a way that it can more readily cross barriers in its free energy landscape.^19,20^ As such, a wide range of techniques have been developed, most of which can be categorized by either heating part or all of the system, or adding a bias potential along some or many coordinates termed collective variables (CVs).^21^ MD simulations combined with enhanced sampling techniques can be used to explore the behavior of a system experiencing a constant or time-varying mechanical force.^4,22^ In much of our work, we have focused on the constant force paradigm, in which case a force applied along a CV such as the end–end distance (*d*_end_) of a protein produces a simple modification to the system’s Hamiltonian
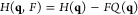
1where *Q*(**q**) is a CV that depends on **q**, the configurational degrees of freedom of the system. The negative sign convention is taken such that a positive *F* corresponds to a pulling force, i.e., which promotes an increase in *Q*.

Motivated by the problem of computing the force–extension behavior of peptides such as disordered formin domains or peptide tension sensors, we previously developed the method Infinite Switch Simulated Tempering in Force (FISST).^23^ There, we demonstrated that it is possible to sample the effect of a range of forces on a system using a single simulation which includes a combination of (a) a special CV-dependent force and (b) an observable weight function that allows one to reweight samples to any intermediate force, as described in the next section. We also demonstrated that FISST can promote transitions between otherwise kinetically inaccessible states of a system due to the action of the additional bias potential. This method was implemented and released as a module in the PLUMED open source sampling library,^24,25^ and we also described its use in a PLUMED masterclass.^1^

However, in some cases, we find that when applying FISST to peptides or proteins for which small forces should result in a population of extended states the system remains trapped near its initial configuration. We therefore wish to combine the efficiency of FISST for sampling many simultaneous forces with a method that is more effective at exploring the conformational states of the molecule.

**Figure 1:**
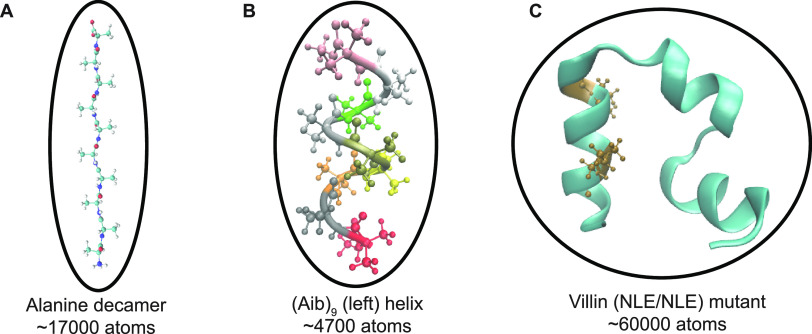
Systems probed in this study, shown without solvent for clarity. (A) Solvated alanine decamer starting in the extended state. (B) Solvated Aib_9_ molecule starting from the left-handed helical state. Each residue is colored according to the residue ID number. (C) Solvated villin (NLE/NLE) mutant starting in the folded state. Locations of residue mutations are colored in ochre. In all cases, pulling forces are applied to the terminal C_α_ atoms.

Here, we demonstrate that the performance of FISST can be improved by coupling it with replica exchange (RE) approaches^19,20,26^ using three benchmark systems of increasing difficulty (Figure 1). After giving a theoretical overview of FISST and how it is naturally coupled with RE, we demonstrate that FISST combined with temperature replica exchange accelerates sampling for our previous test case of an alanine decamer.^23^ We then give the example of the achiral Aib_9_ helical peptide, where FISST alone is not enough to destabilize the folded state, but FISST combined with temperature or solute tempering allows robust sampling of the *F* = 0 free energy landscape and prediction of the force extension curve for this molecule. Finally, we show data computing the force–extension behavior for a more complicated molecule, a villin headpiece mutant; this system is both well characterized in MD simulations and is a variant of a protein whose force–extension behavior has been measured experimentally as a TSM.^10−12,27^

## 2 Theory

### 2.1 FISST Overview

The aim of FISST is to compute averages of observables *O*(**q**) when a constant force *F* is applied along a collective variable *Q*(**q**). At constant temperature, this corresponds to
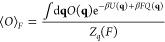
2where β = 1/(*k*_B_*T*), *U* is the potential energy function for the system, and *Z*_*q*_(*F*) ≡ ∫d**q**e^–β*U*(**q**)+β*FQ*(**q**)^ is the configurational partition function for a given *F*.

In ref (23), we showed that averages of this type can be obtained from a single simulation with a modified applied force *F̅*(*Q*) that is derived from the infinitely fast switching limit which would arise if sampling a ladder of applied forces from *F*_min_ to *F*_max_.

In this limit, the probability density that would be sampled is a weighted average over all forces, with weights ω(*F*) that say how important each force is
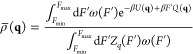
3From this distribution, we get the potential of mean force up to an additive constant through
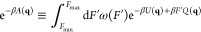
4The FISST algorithm attempts to learn ω(*F*) “on-the-fly” such that forces are sampled evenly, which occurs when ω(*F*) ∝ 1/*Z*_*q*_(*F*), and this is accomplished in an iterative manner. After doing so, the integral of their product becomes a constant *C* ≡ ∫_*F*_min__^*F*_max_^ d*F*′ω(*F*′)*Z*_*q*_(*F*′)

From the potential *A*(**q**) in eq 4, we can get the forces to apply in an MD simulation that will sample from this probability density by taking the negative gradient with respect to atomic positions
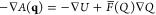
5where
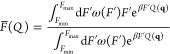
6The FISST module in PLUMED works by computing *F̅*(*Q*) for any choice of CV *Q*, and then modifying the forces used in any compatible MD engine by adding *F̅*(*Q*)∇*Q*. We note that this need not be a simple force/distance pair but could be a more general quantity, e.g., a tension and an area or an electric field and a dipole moment.

After simulating with this modified potential, averages of observables at different forces can be computed from a weighted average over *N*_*t*_ snapshots by including “observable weights” *W*_*F*_(**q**) computed on the fly^23^
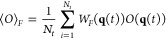
7where
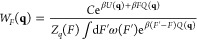
8

### 2.2 Overview of Replica Exchange Methods

In replica exchange simulations, a Markov Chain Monte Carlo procedure is carried out, with detailed balance in exchanges ensuring that each replica maintains a particular equilibrium distribution.^19,20^

In Hamiltonian replica exchange, each replica is simulated via its own Hamiltonian *H*_*i*_, which could be simulated at the inverse temperature β_*i*_. Within each copy of the simulation, configurations appear with probability *P*_*i*_(**q**) ∝ exp(−β_*i*_*H*_*i*_(**q**)).^28^ Ensuring detailed balance of exchange between configurations **q** and **q**′ generated from Hamiltonians *H*_*i*_ and *H*_*j*_, respectively, using a Metropolis criterion requires^28,29^
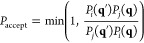
9In this case
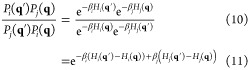
10Below, when we combine FISST with temperature replica exchange (TRE), β_*i*_ will be different for each replica, but we will still be performing a form of Hamiltonian exchange due to the different *F̅*_*i*_(*Q*) computed in each replica. When solute tempering is performed, β_*i*_ will be identical for all replicas; however, in addition to the different bias applied, the potential energy function will also be different between replicas in such a way as to represent effectively higher solute temperatures.

The partial tempering variants that we employ here are based on the Replica Exchange with Solute Tempering (REST) idea.^30−32^ In this work, we use REST3^33^ which scaled interactions between solute and solvent in a way that was shown to not suppress extended configurations of peptides at higher effective temperatures as could occur with earlier REST variants. REST2 and REST3 are formulated such that for *N* replicas, the target temperature of replica *i* is given by , for *i* from 0 to *N* – 1.^32,33^ The potential energy function of each replica *U*_*i*_(**q**) scales the protein–protein and protein–water interactions by factors λ_*i*_^pp^ and λ_*i*_^pw^, respectively.^32,33^ REST3 introduces an additional scaling factor for the nonelectrostatic contributions to the protein–water interactions κ_*i*_. The total potential energy in replica *i* is then given by
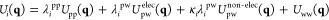
12with λ_*i*_^*pp*^ = *T*_0_/*T*_*i*_, , and κ_*i*_ = 1 + 0.005(*m* – 3)(*m* > 3), with REST2 being recovered if κ_*i*_ is set to unity for all *i*.^32,33^

## 3 Combining FISST with Replica Exchange

Hamiltonian RE is implemented in GROMACS through the PLUMED plugin library.^24,25,29^ In general, PLUMED functions by computing at every step the values of one or several CVs, and then a “bias” energy and forces which is a function of the current CV or CV values. When performing Hamiltonian exchange with GROMACS, PLUMED can use different sets of parameters corresponding to each replica to compute the bias function.^29^ GROMACS computes the force field energy for the original configurations and the swapped configurations. The bias and force field energies are combined, and the total potential energy before and after a proposed swap are compared, with the swap accepted or rejected using eq 11. In this way, very generic replica exchange schemes can be implemented, such as the combination of solute tempering and FISST implemented here.

To enable the combination of FISST with RE, we had to modify our PLUMED implementation such that statistics gathered for computing the quantities ω_*i*_(*F*) and *Z*_*q*_^*i*^(*F*) which are needed for computing the on-the-fly force *F̅*_*i*_(*Q*) are properly computed during the exchange procedure (prior code would update statistics every time the bias is computed, which occurs 3 times during the exchange procedure). This revised code is available from the github page for this paper (see data availability statement) and will be contributed to our FISST module in the public PLUMED library soon.

Computing these quantities using data from the parallel simulations should improve convergence of the weights; however, as discussed in refs (23,34), the observable weights are correct even before these quantities are converged, and in practice, the weights assigned to each force, ω(*F*), can converge quickly so this often does not have a major effect. We also perform simulations where the weights are fixed after an initial equilibration phase.

## 4 Methods

In this section, we provide an overview of the systems studied and simulation protocol. Specific details regarding system setup and simulation parameters are provided in the Supporting Information.

### 4.1 System Details

In this section, we describe the three systems that we will study in this paper. Further simulation data are provided in the Supporting Information.

1. **Ala**_**10**_—This system is the same as that used in our previous FISST study.^23^ In summary, the system consists of a cubic box of size 56.0 Å, solvated using TIP3P water^35^ and parametrized using the CHARMM36 force field. The total system size is 17293, including 5730 water molecules. The system is simulated at 300 K.
2. **Aib**_**9**_—GROMACS^36^ inputs were provided by the authors of ref (37). The system consists of a cubic box of length 35.0 Å, solvated using TIP3P water molecules,^35^ and parametrized using the CHARMM36m force field.^38^ The total size of the system was 4749 atoms including 1540 water molecules. The net charge of the system was neutral with no additional ions added. The system is simulated at 400 K.
3. **Villin Mutant**—Inputs for this system were those generated according to the protocol in ref (39). The 35-residue Villin headpiece “HP35” mutant (PDB ID: 2F4K(40)) was constructed in a cubic box of length 86.80 Å, solvated using TIP3P water molecules,^35^ and parametrized using Amberff99SB*-ILDN force field.^41^ This fast folding mutant has two lysines replaced with the non-natural amino acid norleucine.^40^ The total system size is 60,392 atoms including 19,928 water molecules. The system was neutralized and ions were added to bring the system to a 40 mM salt concentration (15 Na^+^ ions, 16 Cl^–^ ions). The system is simulated at 298 and 360 K. We note that these are the same simulation parameters as described in ref (42) and have also provided those details in our Supporting Information.

### 4.2 Production Runs

#### 4.2.1 Overview

Production data were collected using the GROMACS MD engine.^36^ All single-process MD were run in GROMACS 2020.4, while Hamiltonian exchange simulations were run in GROMACS 2019.6 patched with PLUMED version 2.7.0.^25^ Simulations performed at constant force employed the RESTRAINT feature in PLUMED.^24^

#### 4.2.2 FISST Details

The FISST^23^ algorithm and single-force calculations (applied with the RESTRAINT keyword) were performed using PLUMED.^25^ In all cases, the bias is applied along a collective variable, which is the distance between the first and last C_α_ atoms of the peptides. The FISST force range chosen for Ala_10_ was [−10pN:10pN] and for all other simulations [−10pN:20pN], discretized over 121 gridpoints to perform the integrals.^23^ An initially uniform distribution of the force weights was used. For Aib_9_, weights were updated every 500 steps (1 ps) and both observable and restart data were also saved every 500 steps. For Ala_10_ and HP35, the weights were updated every 1000 steps (2 ps), and the observable and restart data were also saved for the same number of steps.

#### 4.2.3 REST3 Simulations

We implemented the REST3^33^ algorithm for all of our multiple-process MD runs. For Ala_10_, we choose tempering parameters λ and κ parameters using the script provided by ref (33) to simulate a solute temperature range of 300–600 K over 10 replicas, and for Aib_9_ we chose 400–800 K. For Villin mutant, we ran two sets of λ and κ values, with one set of 8 replicas from 298 to 450 K and another set of 8 replicas from 360 to 500 K. Exchanges were attempted every 5 ps. Our REST3 inputs, scripts, and instructions to set up GROMACS topologies for REST3 simulations can be found on the manuscript GitHub (see below).

### 4.3 Data Analysis

All trajectory files were analyzed using the PLUMED driver and mdtraj(43) in Python 3.8.0 Trajectory and structure files were visualized in VMD 1.9.3.^44^

## 5 Results and Discussion

### 5.1 Simulations of Polyalanine Validate Implementation of Hybrid Sampling Approach

In our previous work, we demonstrated using the alanine decamer (Ala_10_) that FISST could accurately compute the end–end distance distribution at a range of forces from a single simulation, as compared to a reference TRE simulation.^23^ Taking this as a stand-in for the more complicated peptides that we wish to probe in the future, we chose this as a benchmark to check that combining FISST with RE does not degrade performance.

Here, we compute the end–end distance probability distribution functions for forces ranging from −10 to 10 pN using a combination of FISST and RE approaches. We first combined TRE and FISST by running 40 parallel FISST simulations at the same temperatures as our reference TRE simulation, using 100 ns for each replica and a force range of [−10pN:10pN]. As benchmarks, we also show previously obtained results for a 500 ns FISST calculation at *T* = 300 K, and TRE calculations at individual forces computed using 40 replicas of 160 ns per window (6.4 μs total simulation time) with temperatures ranging from 300 to 400 K.^23^ For all analyses presented here, we compute results using the bottom replica.

**Figure 2:**
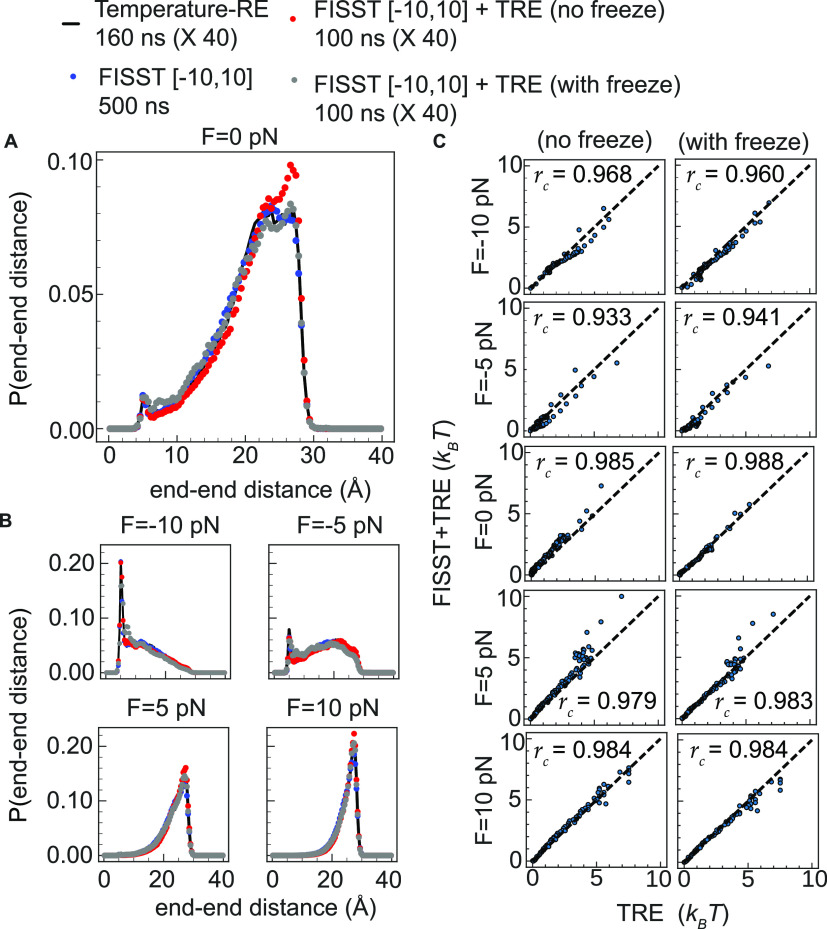
(A) End–end distance distributions for Ala_10_ at *F* = 0 for TRE (black solid line), FISST (blue spheres), FISST + TRE without freezing weights (red spheres), and FISST + TRE with freezing (gray spheres). (B) End–end distributions for *F* = −10, −5, 5, and 10 pN. (C) Comparison of free energies (see Figure S1) computed from end–end distribution functions, comparing FISST + TRE with and without freezing to corresponding reference TRE data at −10, −5, 0, 5, and 10 pN forces.

In Figure 2, we show a comparison of these methods for fixed forces of −10, −5, 0, 5, and 10 pN. We find a reasonable visual agreement from all of our simulation methods relative to our previous FISST and Temperature replica exchange data at all forces, including a peak representing a collapsed state at ∼5 Å for 0, −5, and −10 pN (Figure 2A,B).

However, our initial FISST + TRE run for which we did not freeze the FISST weights shows a slightly higher peak at ∼27 Å compared to the FISST and Temperature RE runs at zero force (Figure 2A, red spheres). We repeated these runs with frozen weights obtained by simulating the parallel replicas without any exchange attempts for 20 ns each and then continued with FISST + TRE with those weights fixed using the FREEZE option in the FISST code. In this case, the data with freezing (Figure 2A, gray spheres) have a more accurate peak at ∼27 Å and better qualitative agreement with the TRE reference.

To check our results quantitatively, we computed the free energy profiles *A*(*Q*) from the probability distribution functions at the different forces shown in Figure 2A,B by taking *A*(*Q*) ≡ – *k*_B_*T* ln(*P*(*d*_end_)) and subtracting an offset such that the minimum in all cases was zero (see Figure S1). We then constructed scatter plots of the two sets of FISST + TRE free energies (with and without freezing the weights) versus the TRE data at each of the corresponding forces, with results shown in Figure 2C. For each of the free energy scatter plots, we computed the Spearman’s rank correlation coefficient^45^ (*r*_c_) using stats.spearmanr function implemented in scipy.^46,47^ Although both sets of data gave relatively high *r*_c_ values when averaged over the 5 forces (0.9698 and 0.9712 without and with freezing the weight distributions, respectively), we observe a slight improvement when freezing the weights for the cases of −5, 0, and 5 pN forces.

**Figure 3:**
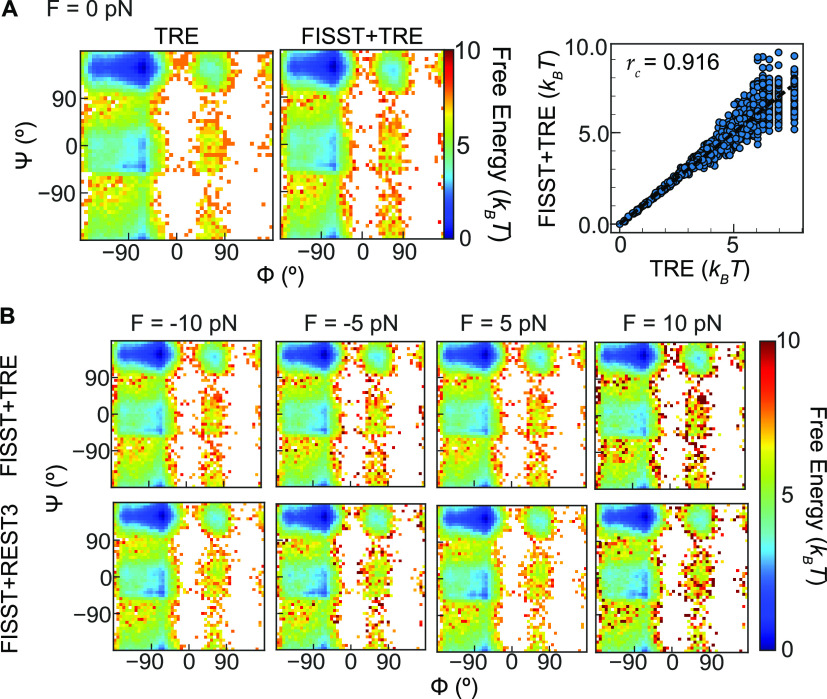
(A) (Left) TRE and FISST + TRE Ramachandran plot at zero force and (right) scatter plot comparing FISST + TRE and TRE free energies at zero force for Ala_10_. (B) Ramachandran plots at −10, −5, 5, and 10 pN forces for FISST + TRE (top row) and FISST + REST3 (bottom row).

We emphasized in our previous work^23^ that using the observable weights (eq 8) we are able to reconstruct averages of other observables at any force besides the one that was biased. In addition to the end–end distance, we also reconstructed Ramachandran plots for Ala_10_ FISST + TRE simulations, reweighting at the zero force shown in Figure 3A. We also computed *r*_c_ between the free energies in both sets of data and found a relatively high value of 0.916. In Figure 3B, we show the result of reweighting the backbone dihedral angles at −10, −5, 5, and 10 pN forces and noted the expected strengthening of the PPII basin (top left) at high force and destabilization of the α-helical basin right center as force is increased.^48^

To demonstrate the accuracy and generality of our implementation, we also performed FISST simulations coupled to REST3 and repeated our end–end distance and Ramachandran angle analysis for the −10, 0, and 10 pN forces using REST3 simulation data collected at those forces. We carried out an initial FISST + REST3 run without freezing the weights and another FISST + REST3 by freezing the weights after 20 ns in an analogous fashion and compared the results of each run to our reference data (Figures S2 and S3). In Figure S2A, we again find reasonable qualitative agreement in the probability distribution functions at all of the forces shown. Qualitative analysis in Figure S2A finds relatively high *r*_c_ values for both FISST + REST3 runs, an average of 0.982 and 0.977 when freezing the weights and without, respectively, indicating a slight improvement compared to the reference REST3 calculations when freezing. We also reweighted the Ramachandran angles calculated from FISST + REST3 (Figure 3B, bottom row) at the forces shown to visually demonstrate that the combination of FISST + REST3 gives equivalent results to FISST + TRE, with quantitative analysis shown in Figure S3.

### 5.2 Simulations of Aib_9_ Show Improved Performance from Hybrid FISST + RE Sampling

While our results on Ala_10_ show that we are able to combine FISST with replica exchange techniques, they do not demonstrate an obvious improvement that requires such a hybrid method. In this section, we show that FISST alone may not be able to sample the free energy landscape of a structured peptide, necessitating the additional sampling from tempering.

Here, we analyze results for the achiral Aib_9_ system starting from a left-handed configuration at *T* = 400 K, for which we initially performed a 4.0 μs unbiased simulation and a 2.0 μs FISST simulation for the force range [−10pN:20pN]. While the unbiased simulation shows a transition rate of approximately 1 inversion per 2 μs (Figure S4), the FISST simulation actually does not, showing a case where FISST can impede conformational exploration. Hence, we felt this is an ideal test system for demonstrating the effectiveness of FISST + RE.

**Figure 4:**
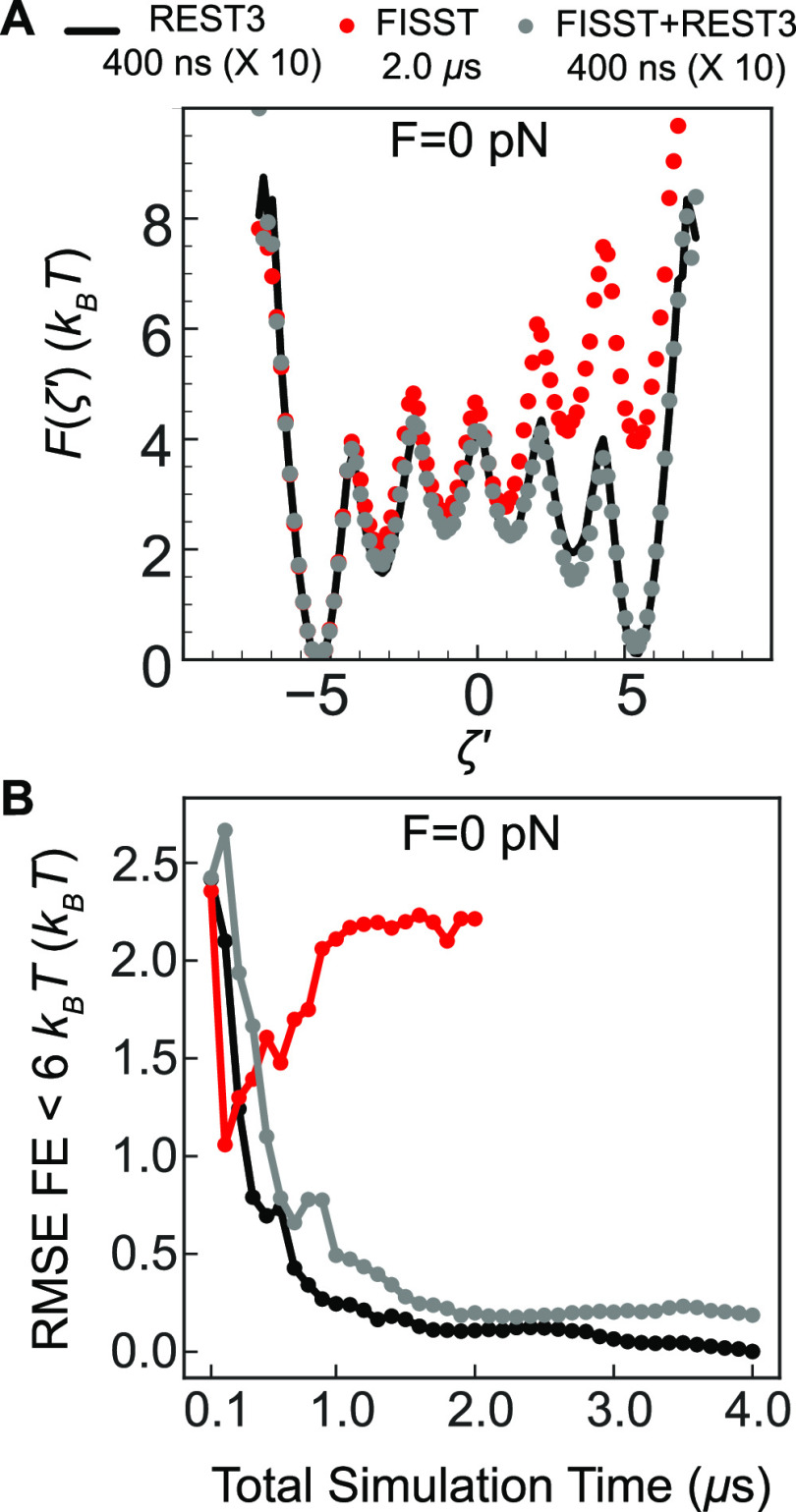
(A) *F*(ζ^′^) at zero force calculated from REST3 (black solid line), FISST (red spheres), and FISST + REST3 (gray spheres). (B) RMSE of *F*(ζ^′^) for values below 6 *k*_B_*T* using data points from different simulation time windows.

We measured the chirality transition of the Aib_9_ helix using the ζ^′^ coordinate defined as the negative sum of the five inner ϕ dihedral angles shown in Figure S4, as done in previous studies.^37,39,49^ With this definition using angles in radians, the left-handed helical configuration takes on a value of ζ^′^ = −5 and the right-handed ζ^′^ = 5. We constructed *F*(ζ^′^) at zero force for each of our simulations using 100 equally spaced windows starting from ζ^′^ = −7.5 to ζ^′^ = +7.5 (Figure 4A). When performing 4 μs (400 ns × 10 replicas) of REST3 simulations spanning 400–800 K, a symmetric free energy profile is obtained (black solid line) from the lowest replica in the ladder, showing that solute tempering is an effective sampling approach for this model problem. This is in contrast to the FISST data (red spheres), which fails to sample the right-helix basin even after 2 μs of simulation time.

We then proceeded to combine FISST with REST3 by using the same 10 replicas but adding FISST sampling in the range [−10pN:20pN]. We find that the FISST + REST3 data (gray spheres) not only overcome the poor sampling from the FISST method alone but also converge with the benchmark REST3 data. Snapshots depicting some molecular configurations observed in this process are shown in Figure S5. To quantify the accuracy of the combined sampling, in Figure 4B, we computed the root-mean-squared error (RMSE) of *F*(ζ^′^) for free energies below 6 *k*_B_*T* (chosen to encompass all of the metastable states based on Figure 4A). We computed the RMSE at *F* = 0 for progressively longer time windows, starting with 100 ns. At short times, the simulation does not adequately sample the entire ζ^′^ = [−7.5,7.5] range, as it remains near the left-handed state, resulting in a high error. While as previously noted, the FISST alone simulation does not converge, the FISST + REST3 converges toward the REST3 reference to less 0.5 kcal/mol (∼0.63 *k*_B_*T* for *T* = 400 K) in approximately 300 ns of total sampling. The same trends hold when using all bins for the RMSE calculation (Figure S6). While the FISST + REST3 curve does not approach zero, this appears to be due to simply finite sampling, resulting in slightly more data in the almost-right metastable state for FISST + REST3 and slightly more data in the almost-left state in the reference calculation.

It should also be emphasized that from a practical point of view, if many processors are available, the FISST + REST3 may be faster in wall clock time than running a single long trajectory, where for example we needed a microsecond or more of Metadynamics simulation to converge a good free energy profile for this system, even with a good reaction coordinate^39^ (see Table S9 for simulation times). We also note that the FISST + REST3 data also contain additional information about all forces from −10 to 20 pN, which we will discuss next, making it much more efficient when this data is needed.

**Figure 5:**
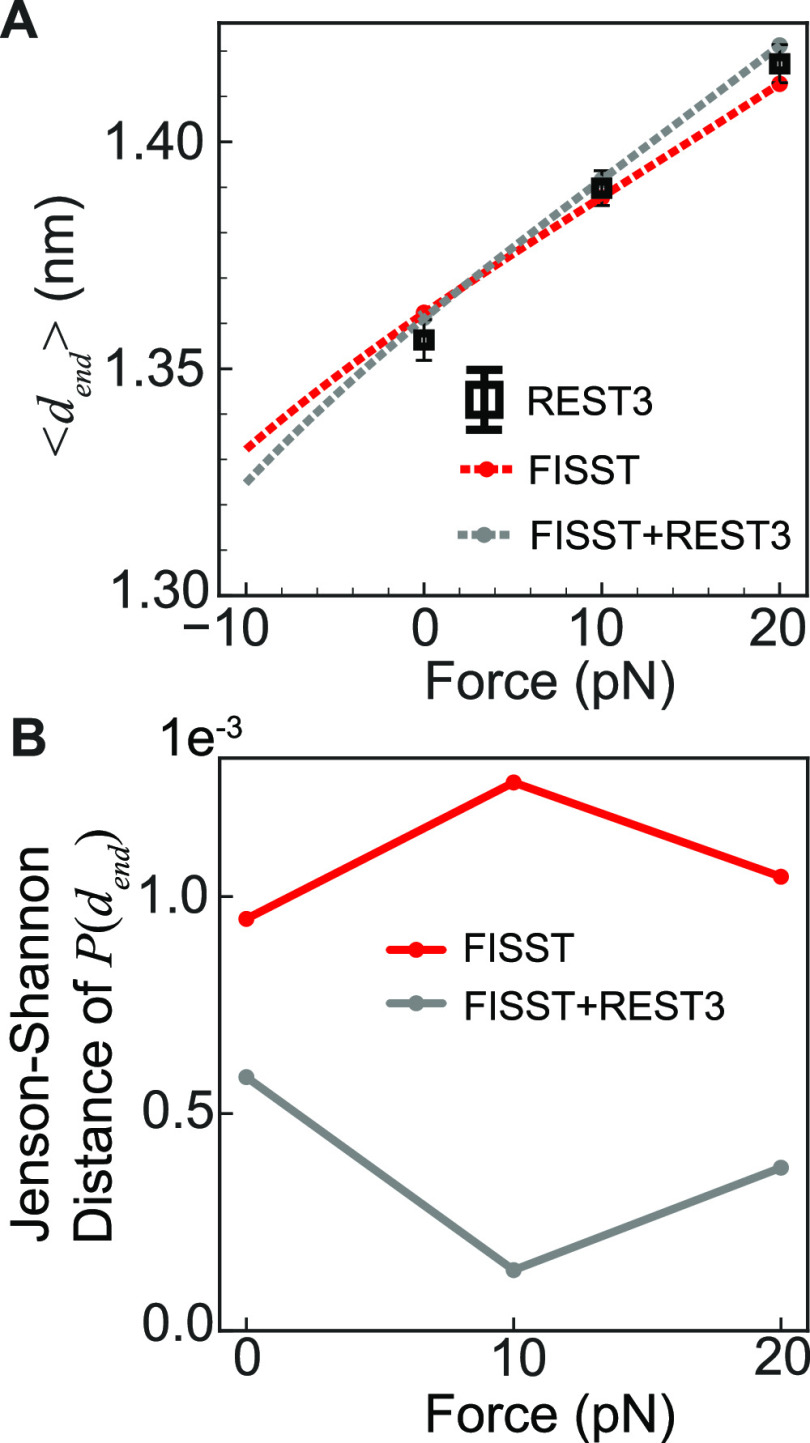
(A) Theoretical force versus average end–end distance ⟨*d*_end_⟩ curve for Aib_9_ calculated from FISST (red dashed line) and FISST + REST3 (gray dashed line). End–end distance values calculated from REST3 simulations at 0, 10, and 20 pN forces (black) and unbiased MD (blue) are embedded for comparison. (B) Jenson–Shannon distance of *P*(*d*_end_) calculated for FISST (red) and FISST + REST3 (gray) for 0, 10, and 20 pN forces using REST3 simulation data as the reference.

In Figure 5A, we show the force extension curve obtained from our simulations by reweighting the end–end distance data to compute a mean distance ⟨*d*_end_⟩ as a function of force. This is an academic exercise since experimental data for this system is not available. Here, we compare the force extension curve for reference REST3 simulations performed at different forces of 0, 10, and 20 pN with our FISST alone or FISST + REST3 simulations. We assess accuracy in Figure 5B as we did in ref (23) by computing the Jenson–Shannon distance^50^ between the reweighted end–end probability distributions. While this analysis shows that our FISST + REST3 result is accurate, it also appears that the FISST alone result is accurate. This is evidently because the Aib_9_ helix is quite resistant to extensional force, and the response is predicted correctly even when trapped in only one helical state.

### 5.3 Villin (NLE/NLE) Mutant Simulations Allow Us to Assess Performance on a TSM-like Molecule

The resistance of Aib_9_ to pulling prevents us from showing the full extent of FISST + REST3’s performance on force extension curves. We now wanted to test our approach for a protein used in a TSM. Many such peptides do not have known structures (because they are not ordered), making it difficult to know if we are using a good starting structure or force field. We therefore decided to study the villin headpiece domain (HP35) since this protein is both well characterized in experiments and probed as a tension sensor module.^27^ Initial test simulations we performed using wild-type HP35 showed little stretching within available simulation time, even with parallel tempering approaches, which we attributed to potential force field overstabilization of collapsed states;^51,52^ force field choice has also been shown to have a very strong effect on the predicted stability of villin in solution.^53^ For this work, we therefore elected to study the Villin (NLE/NLE) mutant whose behavior has been extensively characterized and studied across many simulation studies, and in particular was exhaustively sampled by the DE Shaw Research group.^42^

We analyze data collected from two sets of REST3 simulations consisting of 8 replicas, one with a solute temperature range from 298 to 450 K and another ranging from 360 to 500 K. For each solute temperature range, we ran FISST + REST3 using a force range [−10pN:20pN] for ∼200 ns each (1.6 μs total simulation time). We then repeated these simulations restarting from the point of 20 ns of simulation with weights frozen. We also collected data for *F* = 10 and 20 pN for both solute temperature ranges. An additional reference that we include in our analysis is the ∼310 μs unbiased simulation of Villin (NLE/NLE) at 360 K, identical to the simulation parameters used by the authors of ref (42). Consistent with the analysis of our other systems, we analyze only the bottom replica.

**Figure 6:**
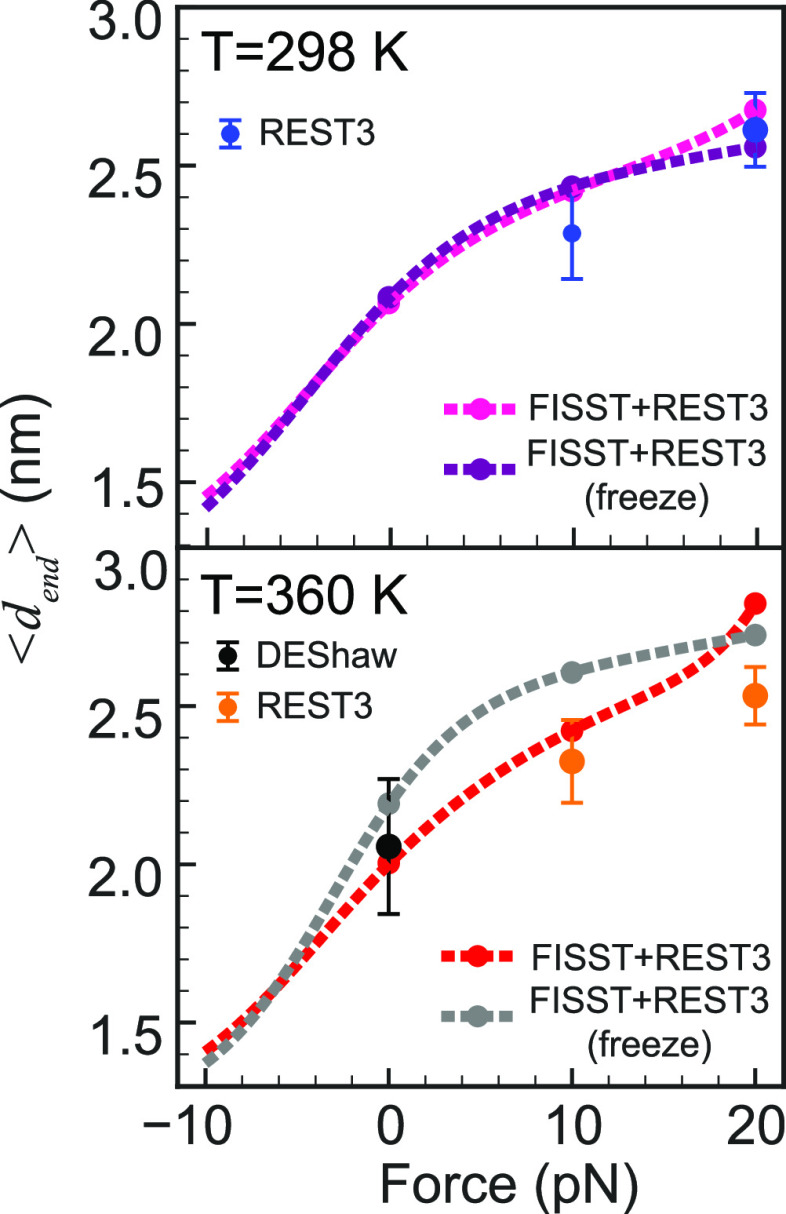
(Top) Villin mutant force vs average end–end distance ⟨*d*_end_⟩ calculated for FISST + REST3 without freezing the weights and freezing the weights at 298 K. ⟨*d*_end_⟩ values calculated from REST3 simulations at *F* = 10 and 20 pN. Error bars represent 1/3 of the standard deviation in length at that force. (bottom). Similar data as above for simulations where the lowest replica is at *T* = 360 K, which is close to the melting temperature. Also shown is data from a 310 μs trajectory at *T* = 360 K from ref (42).

Figure 6 shows our computed force extension curves for the two temperatures selected. The lower is room temperature, where single-molecule pulling experiments are performed on TSMs, and 360 K is close to but below the melting temperature for the mutant using this force field so that many more folding/unfolding events are observed in long unbiased simulations.^42^

Here we observe an elastic regime where the force extension curve is linear from around −5 to +5 pN of pulling force at both temperatures. In both cases, there is a turnover to an inextensible regime, although at each temperature one of the two data sets shows an indication of entering another stretching regime. At *T* = 360 K, the FISST + REST3 curves lie above the reference calculations, which may be an indication of additional sampling of unlikely extended states due to additional sampling from using the hybrid method. We argue that this is due to enhanced sampling rather than hindered sampling because if anything we would naively expect the FISST method to promote spending times at smaller extensions due to the need to sample the full force range from −10 to 20 pN, but the opposite is observed here.

The data shown here contain a discrepancy between simulations of the same length with and without frozen weights. This could be a consequence of the exchange protocol improving the weight calculation such that the FISST calculation in each replica becomes more efficient, resulting in the non-frozen weight data lying closer to the reference single-force results.

In Figure S7, we show how the histograms are transformed as the force on the ends of villin is increased. For both 298 and 360 K, there is a prominent peak at shorter distances (∼1.2 nm) for low force, which is shifted to a prominent peak at larger lengths (∼2.5 nm). The high force distributions are unimodal, although there is some evidence for a shoulder developing at 3.0 nm at the highest forces. The linear increase in average length due to a shift between two populations is something we discussed as the most likely scenario for the low force regime when there are two possible states;^4^ however, here, we are still remaining within compact states, meaning that predominantly unfolded states are not being accessed here. In contrast, experimental data on wild-type HP35 show a full unfolding with a change in length of 7 nm over this force range. For this situation, we previously speculated based on geometric arguments that the force extension curve would have the behavior like we observe here up through ∼5 pN, followed by a separation of the folded state into three independent helices up to around 10 pN, at which point the helices begin to populate fully extended states,^4^ commensurate with the discussion on folded TSMs in ref (11). We hypothesize that our lack of observation of this behavior in the experimentally probed force regime still corresponds to overstabilization of the folded state or collapsed partially unfolded states by the force field/water model.

## 6 Conclusions

In this work, we demonstrated that our force tempering method can be enhanced through a combination with replica exchange approaches. Combination with solute tempering showed a definitive improvement for a test case where FISST alone failed. The combined approach is much more efficient than running many individual simulations at different fixed forces when attempting to compute a full force–extension curve, as in our final example of the HP35 protein. Also, when FISST is combined with TRE, the full force extension profile at all temperatures is obtained simultaneously.

We chose to combine force and temperature sampling by employing a replica exchange approach, which we did because the implementation via a Monte Carlo scheme was practically realizable due to the efforts of the developers of PLUMED and GROMACS.^29,36^ However, we would also like to note that it should be possible to combine infinite switch simulated tempering in force with the infinite switch simulated tempering in temperature (ISST), upon which FISST was originally based.^34^ This may be more effective than our approach here, since at least on paper the infinite switch limit is the most efficient choice for parallel tempering.^34,54,55^ While ISST is implemented in the MIST library,^56^ combining the two approaches would require efficient implementation of estimating partition functions and weights using two-dimensional integrals over both inverse temperature and force which could pose a numerical challenge; hence, we chose not to pursue that effort at this time.

Finally, even with our improved sampling method, we have not yet computed a force extension curve that matches that measured experimentally. While it is possible that the difference is due to the difference in solvent conditions (experiments mostly performed in phosphate-buffered saline or similar), or that the experimental curve is not quite right, given the complex setup using tethering molecules and the need to significantly postprocess data from many pulling runs,^11,27^ for now we presume that the larger error comes from the simulation side. Given that we have implemented and then improved an effective force sampling approach, this points us toward considering alternative water models and protein force fields (or modifying terms in the current ones) to find one that best matches the known behavior for a molecule like HP35. We hope that combining our effective sampling approach with the appropriate force field will allow us to design in silico new tension-sensing peptide molecules.

## Data Availability

All input files, scripts, and output files are available from a GitHub repository for this manuscript, https://github.com/hocky-research-group/FISST-RX_2023. Any additional files will be made available upon request.

## Supporting Information Available

Details of simulation construction; minimization; equilibration; parameters chosen for each method; wall times for Aib_9_ results; free energy profiles along end-to-end distance of the alanine decamer at different forces; Ramachandran plots and errors at different forces for alanine decamer; data from an unbiased simulation of Aib_9_; snapshots of Aib_9_ at different forces and temperatures; data showing convergence of the free energy of Aib_9_ using the full energy range; and end–end distributions at different forces and two temperatures for the villin mutant system (PDF)

## Footnotes

1 Number 22–15, https://www.plumed.org/masterclass
