# Supplementary material for: Improved Prediction of Molecular Response to Pulling by Combining Force Tempering with Replica Exchange Methods
Source: J Phys Chem B. 2024 Jan 17;128(3):706–15. doi: 10.1021/acs.jpcb.3c07081 (PMC10823473; doi:10.1021/acs.jpcb.3c07081)
Supplement: Supplementary file 1 — jp3c07081_si_001.pdf [file jp3c07081_si_001.pdf]

Supporting information for: Improved Prediction of Molecular Response to Pulling by Combining Force Tempering with Replica Exchange Methods

Yuvraj Singh[†] and Glen M. Hocky[*,†,‡]

[†]*Department of Chemistry, New York University, New York, NY 10003, USA*

[‡]*Simons Center for Computational Physical Chemistry, New York University, New York, NY 10003, USA*

E-mail: hockyg@nyu.edu

Simulation Details

System Construction

Table S1: Overivew of system construction details for Alanine decamer (Ala_{10}), Left-handed AIB9 helix (Aib_9), and Villin (NLE/NLE) mutant (HP35). We have also included system setup details for Villin (NLE/NLE) mutant described in Ref. 1, which we use as a reference for our results in Sec. 4.4

	Ala_{10}	Aib_9	HP35	HP35 [Ref. 1]
Forcefield	Charmm36[2]	Charmm36m[3]	Amberff99SB-ILDN[4]	Amberff99SB*-ILDN[4]
Water model	TIP3P[5]	TIP3P[5]	TIP3P[5]	TIP3P[5]
Box size (Å)	56.0	35.0	86.80	54.0
Salt Concentration (mM)	0	0	40	40
Simulated Temperature (K)	300	400	360	360

Minimization

Table S2: System minimization details provided for Alanine decamer (Ala_{10}) and Villin (NLE/NLE) mutant (HP35). No entries for Left-handed AIB9 helix (Aib_9) have been provided as the equilibrated inputs were directly obtained from the authors of Ref. 6.

	Ala_{10}	HP35
Integrator	Steepest Descent	Steepest Descent
Maximum Force (kJ/mol/nm)	1000	1000
Maximum Steps	50000	50000
Nearest Neighbour list type	grid	grid
Cutoff-scheme	Verlet	Verlet
Method for computing long-range Electrostatcs	PME	PME
Short range interactions cutoff (nm)	1	1

Equilibration and Production run inputs

Table S3: Equilibration and Production run parameters used for Alanine decamer (Ala_{10}), Left-handed AIB9 helix (Aib_9), and Villin (NLE/NLE) mutant (HP35).

	Ala_{10}	Aib_9	HP35
Timestep (fs)	2	2	2
Integrator	Leap-frog	Leap-frog	Leap-frog
Thermostat	Berendsen	Nose-Hoover	Berendsen
Barostat	Parrinello-Rahman	Parrinello-Rahman	Parrinello-Rahman
Nearest Neighbour list update frequency	10	20	10
Cutoff-scheme	Verlet	Verlet	Verlet
Method for computing long-range Electrostatcs	PME	PME	PME
Short range interactions cutoff (nm)	1	1.2	1
Algorithm for computing constraints	LINCS	LINCS	LINCS

Production runs

FISST

Table S4: Summary of all single-process simulations with FISST. Force range and total simulation time collected provided for Alanine decamer (Ala_{10}) and Left-handed AIB9 helix (Aib_9). For Aib_9, weights were updated every 500 steps (1 ps) and both observable and restart data were also saved every 500 steps. For Ala_{10}, the weights were updated every 1000 steps (2 ps), and the observable data and restart data were also saved for the same number of steps.

	Ala_{10}	Aib_9
Force range (pN)	[-10,10]	[-10,20]
Total simulation time (μs)	0.5	2
Total number of runs	1	1

REST3

Table S5: Summary of all REST3 simulations without FISST implemented. Forces used, Replica Exchange setup, and total simulation time collected provided for Alanine decamer (Ala_{10}), Left-handed AIB9 helix (Aib_9), and Villin (NLE/NLE) mutant (HP35).

	Ala_{10}	Aib_9	HP35
Solute temperature range (K)	[300,600]	[400,500]	[298,450], [360,500]
Forces (pN)	0, 10, 20	0, 10, 20	0, 10, 20
Number of replicas	10	10	8
Exchange frequency (Number of steps)	2500	2500	2500
Total Simulation Time (μs)	4	4	1.6
Total number of runs	3	3	6

FISST+REST3

Table S6: Summary of all FISST+REST3 simulations without FISST implemented. Force ranges, Replica Exchange setup, and total simulation time collected provided for Alanine decamer (Ala_{10}), Left-handed AIB9 helix (Aib_9), and Villin (NLE/NLE) mutant (HP35). We also specified if we ran additional simulations with the updated PLUMED source code, which freezes the FISST weights. For Aib_9, weights were updated every 500 steps (1 ps) and both observable and restart data were also saved every 500 steps. For Ala_{10} and HP35, the weights were updated every 1000 steps (2 ps), and the observable data and restart data were also saved for the same number of steps.

	Ala_{10}	Aib_9	HP35
Solute temperature range (K)	[300,600]	[400,500]	[298,450], [360,500]
Number of replicas	10	10	8
Exchange frequency (Number of steps)	2500	2500	2500
Force range (pN)	[-10,10]	[-10,20]	[-10,20]
Total Simulation Time (μs)	4	4	1.6
Ran with frozen weights	Yes	Yes	Yes
Total number of runs	2	1	4

Temperature Replica Exchange (TRE)

Table S7: Summary of Temperature Replica Exchange (TRE) performed for Alanine decamer (Ala_{10}). Forces used, Replica Exchange setup, and total simulation time collected are provided.

	Ala_{10}
System temperature range (K)	[300,400]
Number of replicas	40
Exchange frequency (number of steps)	2500
Forces used (pN)	0
Total simulation time (μs)	4
Total number of runs	1

FISST+TRE

Table S8: Summary of FISST+TRE runs performed for Alanine decamer (Ala_{10}). Forces used, Replica Exchange setup, and total simulation time collected are provided. For Ala_{10}, the weights were updated every 1000 steps (2 ps), and the observable data and restart data were also saved for the same number of steps.

	Ala_{10}
System temperature range (K)	[300,400]
Number of replicas	40
Exchange frequency (number of steps)	2500
Forces range (pN)	[0,10]
Total simulation time (μs)	4
Ran with frozen weights	Yes
Total number of runs	2

Alanine Decamer

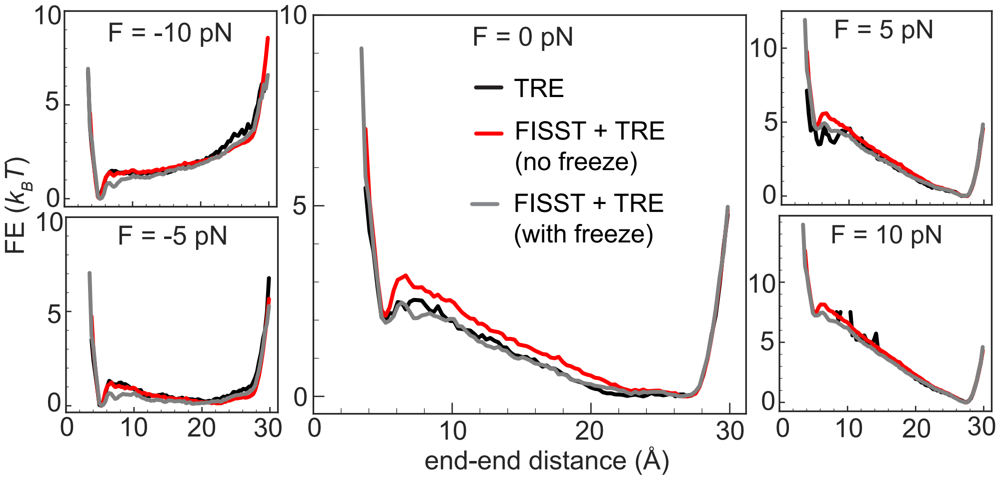

Figure S1: Alanine Decamer Free energy profiles for TRE (black solid line), FISST+TRE without freezing weights (red solid line), and FISST+TRE with freezing weights (gray solid line) calculated from the end-end distance probability distributions at -10, -5, 0, 5, and 10 pN forces shown in Fig. 2(A) and Fig. 2(B).

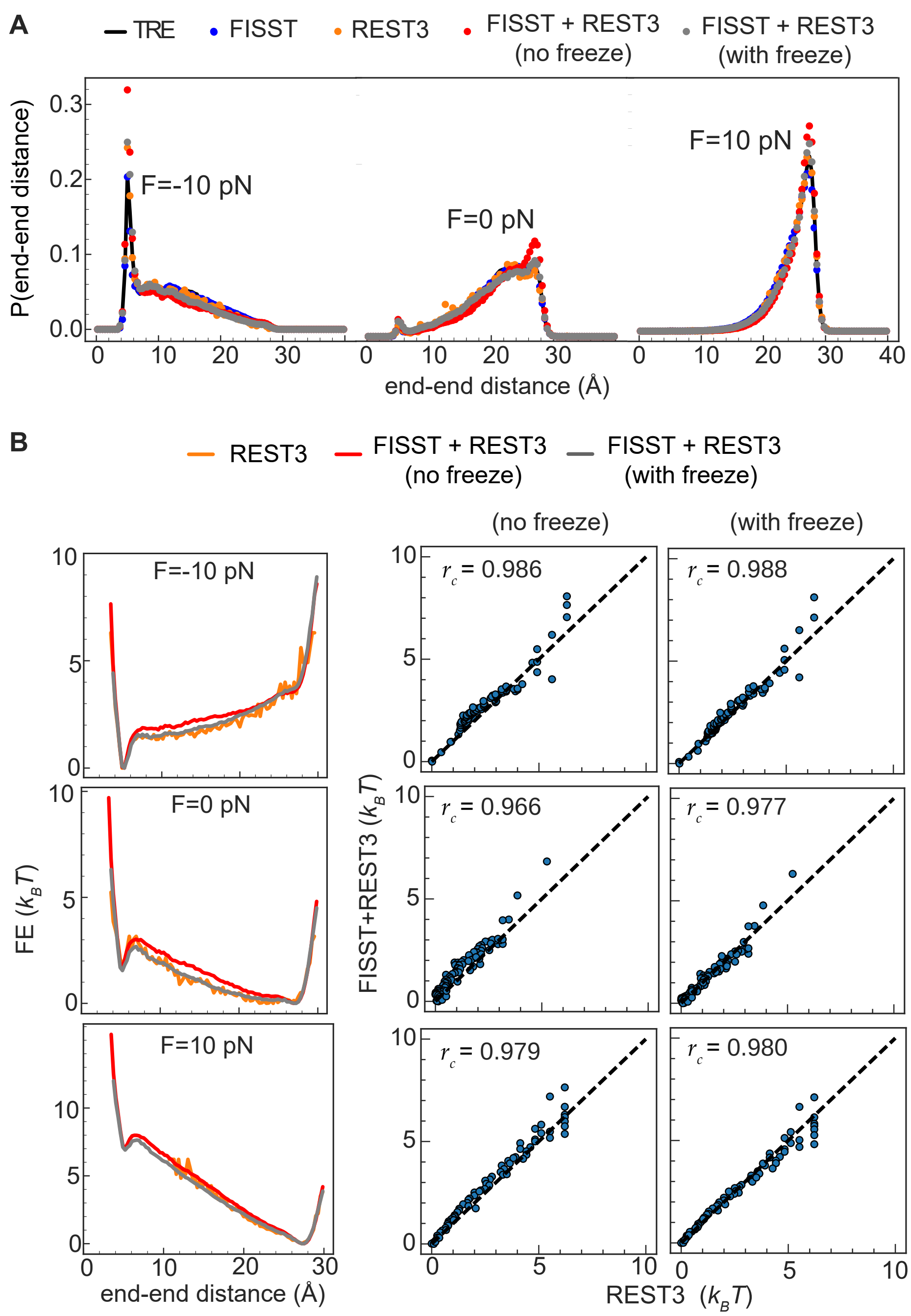

Figure S2: (A) Alanine decamer end-end distance probability distribution functions calculated for TRE (black solid line), FISST (blue spheres), REST3 (orange spheres), FISST+REST3 without freezing the weights (red spheres), and FISST+REST3 with freezing the weights (gray spheres) at -10, 0, and 10 pN forces. (B) (left) Corresponding free energy profiles. (right) Free energy scatter plots comparing FISST+TRE and TRE data without and with freezing of the weights.

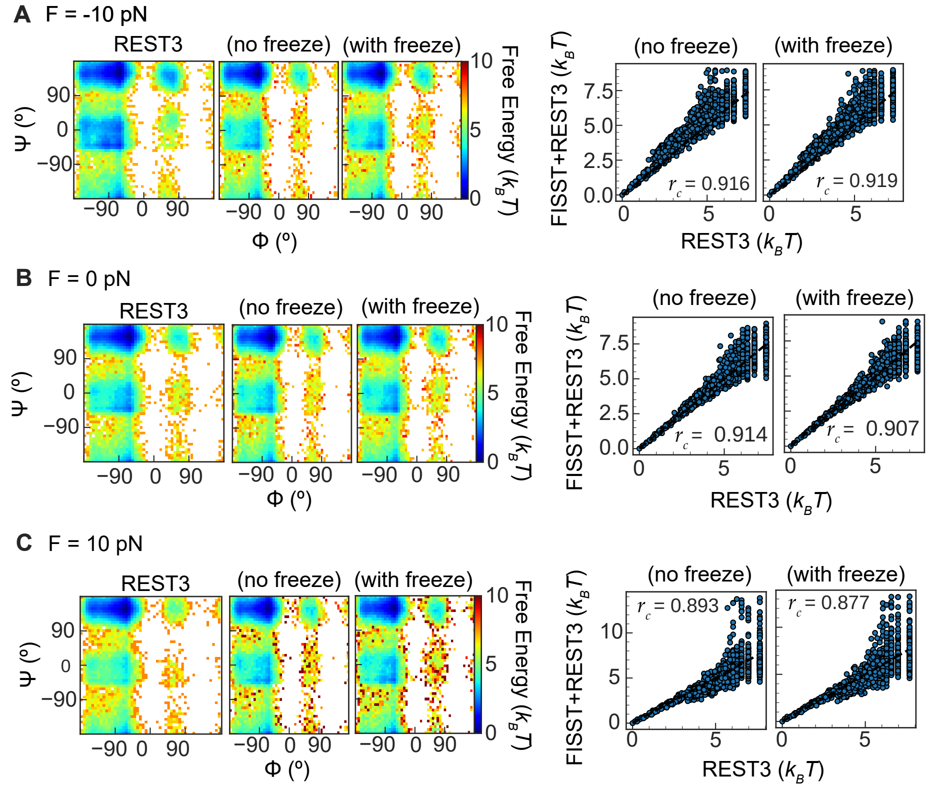

Figure S3: (left to right) Ramachandran plots calculated from REST3, FISST+REST3 without freezing the weights, FISST+REST3 with freezing the weights and corresponding free energy scatter plots comparing FISST+REST3 without and with freezing of the weights for (A) -10, (B) 0, and (C) 10 pN forces.

Aib$_9$

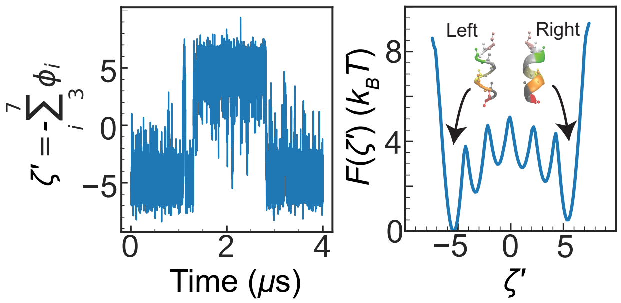

Figure S4: (left) Time series plot for ζ' coordinate generated from unbiased MD simulation of Aib$_9$. (right) Corresponding free energy profile of ζ'. (inset) Left and right-handed Aib$_9$ helices are marked in their respective basins.

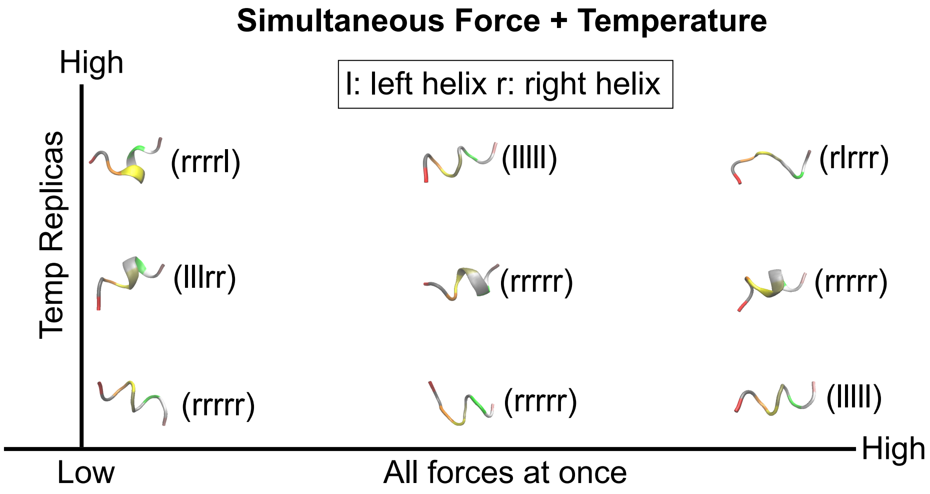

Figure S5: Snapshots from Aib$_9$ FISST+REST3 trajectory depicting helical compositions at different forces and solute temperature replicas.

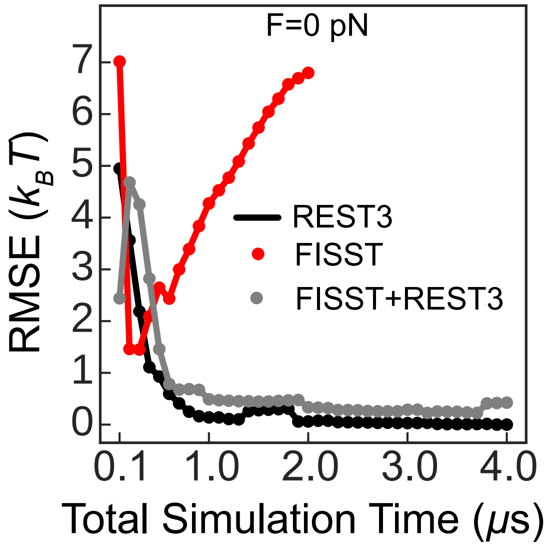

Figure S6: RMSE of $F(\zeta')$ at zero force calculated from full free energy profiles from REST3 (black), FISST (red), and FISST+REST3 (gray) using data points from different simulation time windows.

Table S9: Summary of total simulation and wall times of all Aib_9 runs.

	Simulation Time (μs)	Wall time (days:hours)
Unbiased MD	4	14:19
FISST (single MD)	2	8:9
RE ($\times$10)	4	1:16
FISST+RE ($\times$10)	4	1:13

Villin (NLE/NLE) Mutant

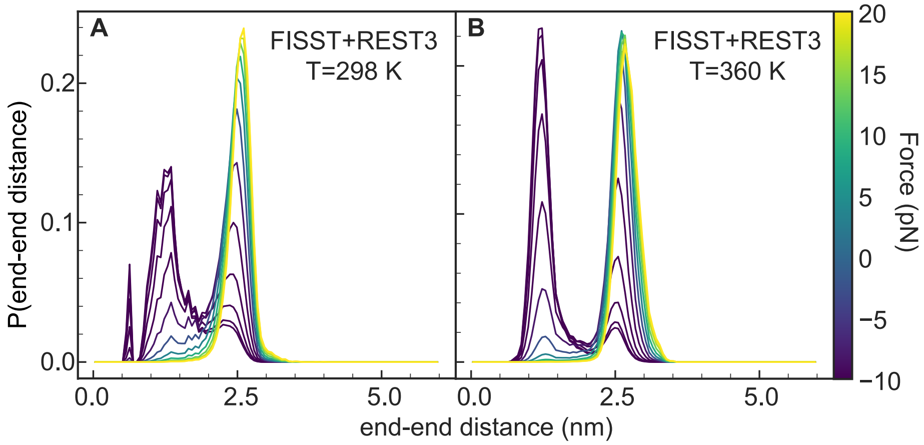

Figure S7: Villin Mutant end-end distance probability distribution reweighted to forces in the range [-10 pN:20 pN] from FISST+REST3 simulations at (A) Solute temperature of 298K and (B) Solute temperature of 360K. The color bar distinguishes the forces to which the distributions are reweighted.

References

(1) Piana, S.; Lindorff-Larsen, K.; Shaw, D. E. Protein folding kinetics and thermodynamics from atomistic simulation. *Proc. Natl. Acad. Sci.* **2012**, *109*, 17845–17850.

(2) Best, R. B.; Hummer, G. Optimized molecular dynamics force fields applied to the helix-coil transition of polypeptides. *J. Phys. Chem. B* **2009**, *113*, 9004–9015.

(3) Huang, J.; Rauscher, S.; Nawrocki, G.; Ran, T.; Feig, M.; De Groot, B. L.; Grubmüller, H.; MacKerell Jr, A. D. CHARMM36m: an improved force field for folded and intrinsically disordered proteins. *Nat. Methods* **2017**, *14*, 71–73.

(4) Lindorff-Larsen, K.; Piana, S.; Palmo, K.; Maragakis, P.; Klepeis, J. L.; Dror, R. O.; Shaw, D. E. Improved side-chain torsion potentials for the Amber ff99SB protein force field. *Proteins: Struct. Func. Bioinf.* **2010**, *78*, 1950–1958.

(5) Jorgensen, W. L.; Chandrasekhar, J.; Madura, J. D.; Impey, R. W.; Klein, M. L. Comparison of simple potential functions for simulating liquid water. *J. Chem. Phys.* **1983**, *79*, 926–935.

(6) Mehdi, S.; Wang, D.; Pant, S.; Tiwary, P. Accelerating all-atom simulations and gaining mechanistic understanding of biophysical systems through state predictive information bottleneck. *J. Chem. Theor. Comput.* **2022**, *18*, 3231–3238.
